# Navigating epigenetic epidemiology publications

**DOI:** 10.1186/s43682-023-00023-3

**Published:** 2023-11-22

**Authors:** Wei Yu, Emily Drzymalla, Matheus Fernandes Gyorfy, Muin J. Khoury, Yan V. Sun, Marta Gwinn

**Affiliations:** 1 Office of Genomics and Precision Public Health, Centers for Disease Control and Prevention, Atlanta, GA, USA; 2 Department of Epidemiology, Rollins School of Public Health, Emory University, Atlanta, GA, USA; 3 Tanaq Support Services, Atlanta, GA, USA

**Keywords:** Epigenetic, Epidemiology, Database

## Introduction

Since its beginning more than 75 years ago [1], epigenetics has been an evolving field with growing applications to the study of cancer, aging, and gene expression in response to environmental exposures. The emergence of high-throughput technology for measuring epigenetic markers has enabled population-based studies [2]. The relatively new field of epigenetic epidemiology investigates epigenetic associations from a population perspective for insights into disease risk, prevention, and progression. Unlike genetic variants, epigenetic markers are dynamic, offering epidemiologists a new approach to linking early life and environmental exposures with disease risk [3].

Scientific publications on epigenetic epidemiology have been rapidly increasing in number and variety over the past 20 years. The literature now includes studies of epigenetic markers beyond DNA methylation (DNAm), such as histone modification and non-coding RNA, and consists of a variety of study designs including epigenome-wide association studies (EWAS), candidate gene studies, and clinical trials. Epigenetic markers are investigated as risk factors, such as DNAm in association with type 2 diabetes incidence [4], or outcomes, such as DNAm changes in response to air pollution [5]. The objective of the Epigenetic Epidemiology Publications Database (EEPD) is to offer a user-friendly website to explore the expanding literature in epigenetics, epidemiology, and public health.

## Methodology

In order to better navigate this literature, we created the Epigenetic Epidemiology Publications Database (https://phgkb.cdc.gov/PHGKB/eEStartPage.action). We aimed to include population-based studies of epigenetics in association with an exposure or outcome of interest, systematic reviews related to epigenetic associations, and published descriptions of epigenetic resources such as cohorts, protocols, and methods. We aimed to exclude articles on gene expression (e.g., transcriptomics), non-population-based laboratory studies, non-human studies, and non-systematic reviews. We developed an automatic screening process that starts with a PubMed query, followed by application of a machine learning method (support vector machine, SVM) combined with text mining techniques [6]. We assessed the performance of our screening process using a random sample of 1999 manually annotated abstracts. Of these, 285 positive and 114 negative records were used for testing. We estimated area under the curve (AUC) at 96.4% with 90% sensitivity and 90% specificity.

To build the database, records are automatically selected and uploaded daily. Each record is indexed with genes, diseases, and other factors. The system also automatically subsets the data according to 14 special topics (cancer, diabetes, economic evaluation, environmental health, family health history, health equity, heart, lung, blood and sleep disorders, infectious diseases, neurological disorders, pharmacogenomics, primary immune deficiency diseases, rare diseases, and reproductive and child health) by searching the database with specially topic relevant terms.

## Description of database

EEPD is a publicly available, web-based application with a user-friendly web interface designed to provide easy access to the scientific literature of epigenetic epidemiology. EEPD provides the opportunity to search for epigenetic epidemiology related literature without having to form a complicated PubMed query. For example, on September 28, 2023, a search for cancer-related epigenetic epidemiology on EEPD provided 8942 records, while a search on PubMed using the terms “cancer” and “epigenetics” and “epidemiology” provided 1198 records, and removing the “epidemiology” term resulted in 17,927 records. Users can type the query of interest in the search box on the top of the page, and the search result will be returned as a list of publications related to the query. To further refine the returning result, the user can use the filtering functions including the following indexed factors such as gene, disease, publication year, publication journal, and study design. The filtering function can be performed continually until the desired result is returned. The final search results can be downloaded as an Excel spreadsheet by clicking on the “Download” button. The database also can be subset to a specific special topic by selecting a dataset for a topic of interest in the dropdown menu in the search bar. With the subset, all the navigation functions can be performed as above.

**Fig. 1:**
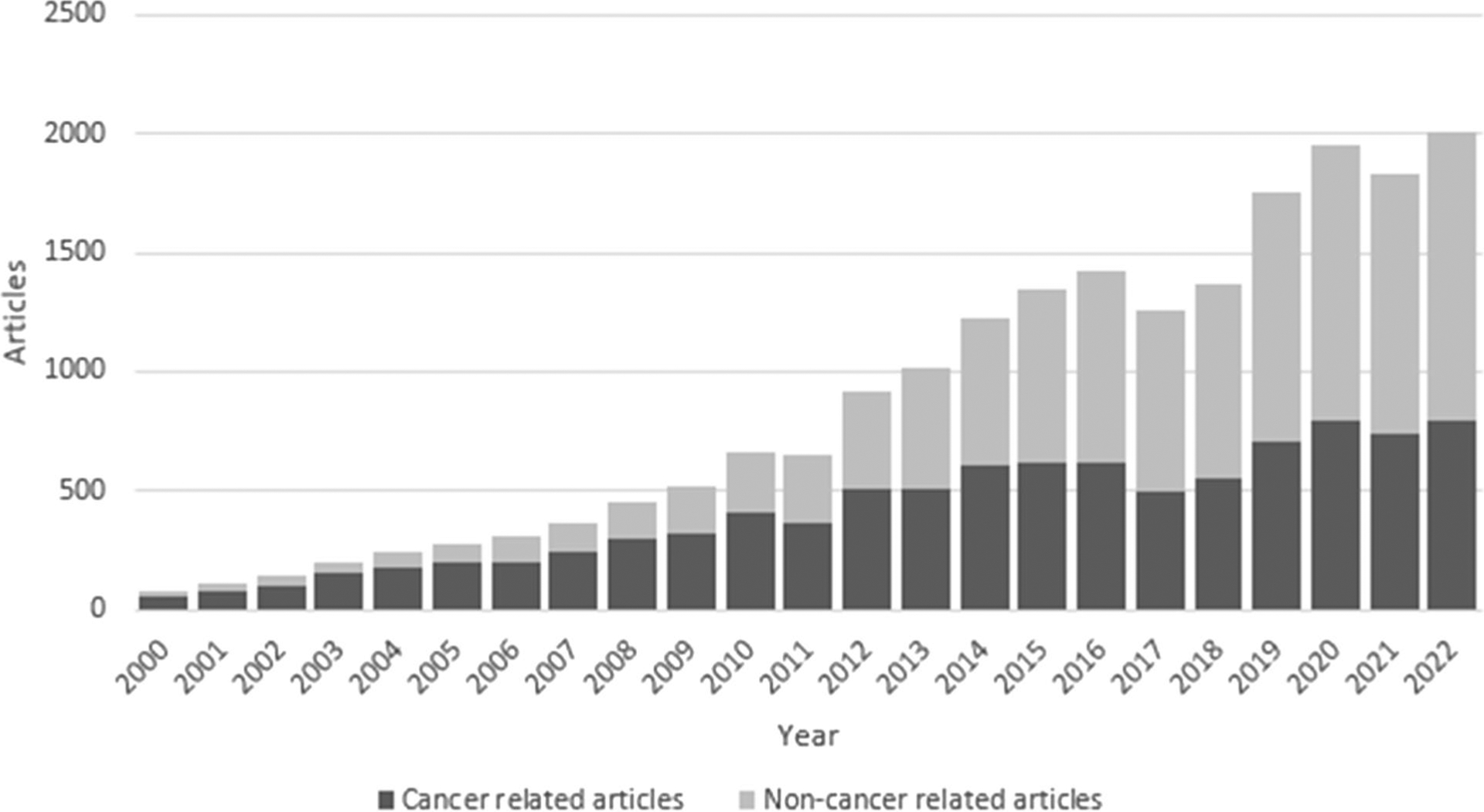
Epigenetic Epidemiology Publications Database articles per year

As of August 18, 2023, the database contains 21,699 PubMed records, indexed with 9378 genes and 1019 disease terms. Since 2000, the annual number of publications has increased markedly (Fig. 1). The most studied topic is Cancer and followed by Rare Diseases. The 5 most frequently indexed disease terms are breast neoplasm, colorectal neoplasms, squamous cell carcinoma, lung neoplasm, and stomach neoplasms, while the 5 most frequently indexed genes are CDKN2A, RASSF1, DNMT1, MGMT, and CDH1.

## Conclusion

EEPD is a novel tool for navigating the epigenetic epidemiology literature, expediting the search for information at the intersection of epigenetics, epidemiology, and public health without labor-intensive screening. EEPD offers investigators an efficient way to gain an overview of available research on a particular topic as well as to find articles relevant to their own projects. The sensitivity of EEPD is not perfect. The search algorithms and indexing processes of PubMed and EEPD have their own limitations in a field that is rapidly evolving and not fully defined. Furthermore, because EEPD relies exclusively on PubMed as a data source, potentially relevant articles not indexed there are not included. Despite these limitations, EEPD can be complementary to traditional search methods for literature review.

In the future, we would hope to expand the data sources for EEPD, using resources such as Scopus and Embase. Further tuning the PubMed search query and EEPD algorithms could also enhance the sensitivity and specificity of data collection and retrieval.

## Data Availability

All data in Epigenetic Epidemiology Publications Database can be accessible via the following URL: https://phgkb.cdc.gov/PHGKB/eEStartPage.action
